# Rapid diagnosis of acute norovirus-associated gastroenteritis: evaluation of the Xpert Norovirus assay and its implementation as a 24/7 service in three hospitals in Jönköping County, Sweden

**DOI:** 10.1007/s10096-017-3005-9

**Published:** 2017-05-24

**Authors:** A. J. Henningsson, A. Nilsson Bowers, J. Nordgren, M. Quttineh, A. Matussek, S. Haglund

**Affiliations:** 1 Clinical Microbiology, Laboratory Medicine, Region Jönköping County, S-551 85 Jönköping, Sweden; 2 Medical Microbiology, Department of Clinical and Experimental Medicine, Linköping University, Linköping, Sweden; 3 Karolinska University Laboratory, Karolinska University Hospital, Solna, Sweden; 4 Division of Clinical Microbiology, Department of Laboratory Medicine, Karolinska Institute, Karolinska University Hospital, Huddinge, Sweden

## Abstract

Noroviruses are a leading cause of epidemic and sporadic cases of acute gastroenteritis worldwide. The rapid diagnosis of norovirus infection is important for prompt infection control measures and may reduce the need for additional diagnostic testing. Here we evaluated the performance of the rapid Xpert Norovirus assay, and assessed the turn-around time (TAT) before and after the implementation of the analysis as a 24/7 service at all the three hospitals in Jönköping County, Sweden. We describe the implementation process which was performed in two steps during 2014. A total number of 276 clinical samples (stool and vomitus) from patients with symptoms of acute gastroenteritis were included in 2014–2015. The samples were analysed with the Xpert Norovirus assay and the already existing routine method: an in-house reverse transcription real-time PCR. Samples showing discrepant results with the two assays were further analysed by a third PCR method. The Xpert Norovirus assay performed well with a sensitivity of 100% and a specificity of 93% compared to the gold standard (defined as the result obtained by at least two of the three PCR methods). The median TAT decreased from 22 hours in 2013 to 2.4 hours in 2015 (p<0.001). We conclude that the performance of the Xpert Norovirus assay was excellent, and that the implementation of the analysis as a 24/7 service at all three hospitals in the county has greatly reduced the time to diagnosis which is beneficial for both patients and healthcare providers.

**Electronic supplementary material:**

The online version of this article (doi:10.1007/s10096-017-3005-9) contains supplementary material, which is available to authorized users.

## Introduction

Noroviruses (NoV) are highly infectious non-enveloped RNA viruses and a leading cause of acute gastroenteritis worldwide [1]. There are seven known genogroups, designated genogroup I (GI) to GVII, and over 40 genotypes [2]. The GI and GII are the most important for human infection [3, 4]. NoV infections are typically self-limiting, but may cause severe disease in the most vulnerable, i.e. immunocompromised persons, elderly and small children [5]. NoV is easily transmitted in semi-closed units, such as hospitals and senior care facilities. Rapid and reliable laboratory diagnostics for early identification of outbreaks and sporadic cases are essential for prompt infection control measures and prevention of nosocomial spread [6–10].

Laboratory methods for detection of NoV in clinical samples have evolved over time, and nucleic acid amplification tests (NAAT) have now become the mainstay [11]. Stool is generally regarded as the sample of choice due to the higher viral load as compared to vomitus.

The annual number of NoV samples in Jönköping County, Sweden, is around 1300, and NoV diagnostics used to be centralized at the clinical microbiology laboratory (CML) at the County Hospital Ryhov, Jönköping. The analysis, an in-house one-step reverse transcription (RT) real-time PCR based on Kageyama et al. [12], was performed once to twice daily, and analyses were delayed by transportation and batching of samples. The Xpert® Norovirus assay, a NAAT for the GeneXpert instrument (Cepheid, Sunnyvale, CA, USA), is a single-unit rapid easy-to-use test which can be run on demand and requires little hands-on time. Therefore, the analysis could, after an initial evaluation, be decentralized from the sole CML (open 7.30 a.m. to 7.00 p.m.) to the clinical chemistry laboratories (CCLs) (open 24 h a day, every day of the week [24/7]) at the three hospitals in our county: Jönköping, Eksjö and Värnamo.

The aim of this study was to evaluate the diagnostic accuracy of the Xpert Norovirus assay and to assess the median turn-around time (TAT) before and after the implementation of the analysis as a 24/7 service.

## Materials and methods

### Implementation of the Xpert Norovirus assay at all three hospitals

After an initial evaluation at the CML, GeneXpert instruments and the Xpert Norovirus assay were set up at the CCLs at all three hospitals in the county during spring 2014. Education of the staff and local validation of the analysis was performed by specialists in molecular diagnostics, who also kept the responsibility for the method’s accuracy and the follow-up of external quality controls. Implementation in clinical routine was performed in two steps. From May to November 2014, the analysis was performed daytime in the CCLs in Eksjö and Värnamo, whereas the analysis remained at the CML in Jönköping in order to monitor and identify unexpected problems. During November 2014, the analysis was implemented as a 24/7 service at all three CCLs in the county.

### The GeneXpert and the routine method in parallel

Following the initial evaluation, samples were continuously analyzed in parallel at the CCLs and at the CML in order to follow the quality and performance of the Xpert Norovirus assay in clinical practice. A total number of 276 samples (stool, *n* = 257; vomitus, *n* = 19) from patients with symptoms of acute gastroenterits were included in the study in 2014–2015.

### Xpert Norovirus assay with GeneXpert

All samples were prepared and analyzed with the Xpert Norovirus kit on GeneXpert at the CCLs according to the instructions from the manufacturer. Briefly, a small amount of feces or vomitus was transferred to a vial of sample reagent, vortexed and transferred to the assay cartridge. A sample processing protocol and a probe check control were contained in each cartridge and analyzed in conjunction with each sample. Each cartridge detected NoV GI and GII simultaneously with hydrolysis probes. Samples were interpreted as positive or negative based on their threshold cycle (C_t_ value) and endpoint signal, via an algorithm in the GeneXpert software, and the presented results included detected GI or GII.

### Routine in-house method

The in-house one-step RT real-time PCR for detection of NoV GI and GII was based on Kageyama et al. [12], with the following modifications: the probe for detection of NoV GII (RING2-TP) was labeled with LC670 to allow multiplex PCR and detection, and a BlackBerry quencher was used instead of Tamra. BlackHole quenchers were used on RING1(a) and RING1(b) for detection of NoV GI (TibMolBiol, Berlin, Germany). RING1(a) was used at 6 pmol, RING1(b) and RING2-TP were used at 2 pmol. Briefly, RNA was extracted from 10 μL feces dissolved in 300 μL H_2_O or from 300 μL of liquid samples. After a brief centrifugation of the samples, viral RNA was extracted using the MagAttract Viral RNA M48 kit (Qiagen, Hilden, Germany) with the BioRobot M48 according to the manufacturer’s instructions. One-step RT real-time PCR was performed with 5 μL of template in a final reaction volume of 20 μL using the LightCycler RNA Amplification HybProbe kit and a LightCycler 480 (Roche, Applied Science, IN, USA). Thermocycling was performed using the following conditions: 55 °C for 10 min, followed by 95 °C for 30 s, 45 cycles at 95 °C for 15 s and 56 °C for 45 s. Positive and negative controls were included in each run and manual analysis of the amplification curves was performed.

### Samples with discrepant results

Samples showing discrepant results with the two assays were further analyzed by a third PCR method modified from Nordgren et al. [13, 14]. Viral RNA was extracted from 300 μL of (10%*v*/*W*) fecal supernatant using the MagAttract Viral RNA M48 kit (Qiagen) as described above. Four μl of purified RNA was added to a reaction mixture consisting of 10 μl of iTaq universal probes reaction mix (BioRad, Stockholm, Sweden), 0.8 μL (10 pmol/μL) of each GI and GII primers (NVG1f1b and NVG1rlux, NVG2flux1 and COG2R), and 0.4 μl (10 pmol/μL of GI and GII probes, 0.5 μL of iScript advanced reverse transcriptase and 1.5 μL of RNAse free water, to a final volume of 20 μl. The one-step RT real-time PCR reactions were performed in a 96-well reaction plate using the CFX96 Real-Time PCR Detection System (BioRad). The RT real-time PCR was performed under the following conditions: 50 °C for 10 min followed by 95 °C for 3 min and 45 cycles of 95 °C for 5 s, and 60 °C for 30 s.

### Turn-around time

The median TAT was measured before (2013) and after (2015) the GeneXpert® Norovirus assay was implemented as a 24/7 service at all three hospitals in the county. TAT was measured from the time point that the sample was sent from the healthcare unit to the time point when the laboratory reported the result. The percentage of test results reported within 4 h was measured from the time point that the sample arrived at the laboratory to the time point when the result was reported. The data were retrieved from DivePort, version 7.0 (Dimensional Insight Inc., Burlington, MA, USA).

### Definition of the gold standard

The gold standard test result was defined as the result obtained by at least two of the following methods: the Xpert Norovirus assay, the in- house PCR method based on Kageyama et al. [12], and the PCR method based on Nordgren et al. [13, 14].

### Statistics

For group comparisons, the Mann-Whitney U-test was used. Data are expressed as median. Statistical analyses were performed using Statistica version 12.7 (StatSoft Inc., Tulsa, OK, USA). *P*-values <0.05 were considered significant.

## Results

### Comparison between the Xpert Norovirus assay and the gold standard

The results from the comparison between the Xpert Norovirus assay and the gold standard test results are presented in Table 1. The sensitivity of the Xpert Norovirus assay was 100% and the specificity was 93%. The positive predictive value was 87% and the negative predictive value 100%. Using the Xpert Norovirus assay, 12 samples were positive for GI, and 85 for GII, whereas according to the gold standard, nine samples were positive for GI and 75 for GII. The positive and negative agreement between the Xpert Norovirus assay and the gold standard was 95%.

**Table 1:** Comparison between the gold standard test result^a^ and the Xpert Norovirus assay for detection of noroviruses (NoV)

Method of detection		Gold standard^a^		
		Positive	Negative	Total
Xpert NoV	Positive	84	13	97
	Negative	0	179	179
	Total	84	192	276

^a^Gold standard: the result obtained by at least two of the three following methods for detection of NoVs: the Xpert Norovirus assay, the in-house method based on Kageyama et al. [12], and the method based on Nordgren et al. [13, 14].

There were 18 samples (stool, *n* = 17; vomitus, *n* = 1) with discrepant results between the Xpert Norovirus assay and the in-house PCR; all of them showing positive test results with the Xpert Norovirus assay (GI, *n* = 6; GII, *n* = 12), and negative with the in-house PCR. When these 18 samples were analyzed with the third PCR method [13, 14], four showed positive results (GI, *n* = 3; GII, *n* = 1). The mean C_t_ value for the 14 samples that had positive results only in the Xpert Norovirus assay was 35.5 (range, 30.4–40.0). Both the in-house PCR and the Xpert Norovirus assay were able to detect NoV in both stool and vomitus.

### Effects on turn-around time

The overall median TAT from arrival of the samples to the laboratories to available test results decreased from 22 h in 2013 to 2.4 h in 2015 (*p* < 0.001) (Table 2). For samples taken at primary healthcare centers, the median TAT decreased from 38 h to 14 h (*p* < 0.001, data not shown).

**Table 2:** The median turn-around time (TAT) before and after the GeneXpert® Norovirus assay was implemented at the laboratories of clinical chemistry as a 24/7 service at all three hospitals in the county

Setting	2013		2015		P-value
	TAT; hours (n)	IQR	TAT; hours (n)	IQR	
All clients	22 (1124)	14–30	2.4 (1289)	2.0–4.2	***
All clients excl. PHC	20 (933)	11–26	2.3 (1123)	2.0–3.2	***
Jönköping	19 (583)	7.8–25	2.4 (778)	2.0–3.4	***
Eksjö	20 (140)	14–26	2.0 (161)	1.9–2.5	***
Värnamo	24 (194)	19–29	2.2 (195)	2.0–3.6	***
PHC only	38 (191)	27–48	14 (166)	6.4–22	***

*PHC* primary healthcare centre, *TAT* median turn-around time from sampling to available test result, *IQR* interquartile range

*** *P* < 0.001

**Fig. 1:**
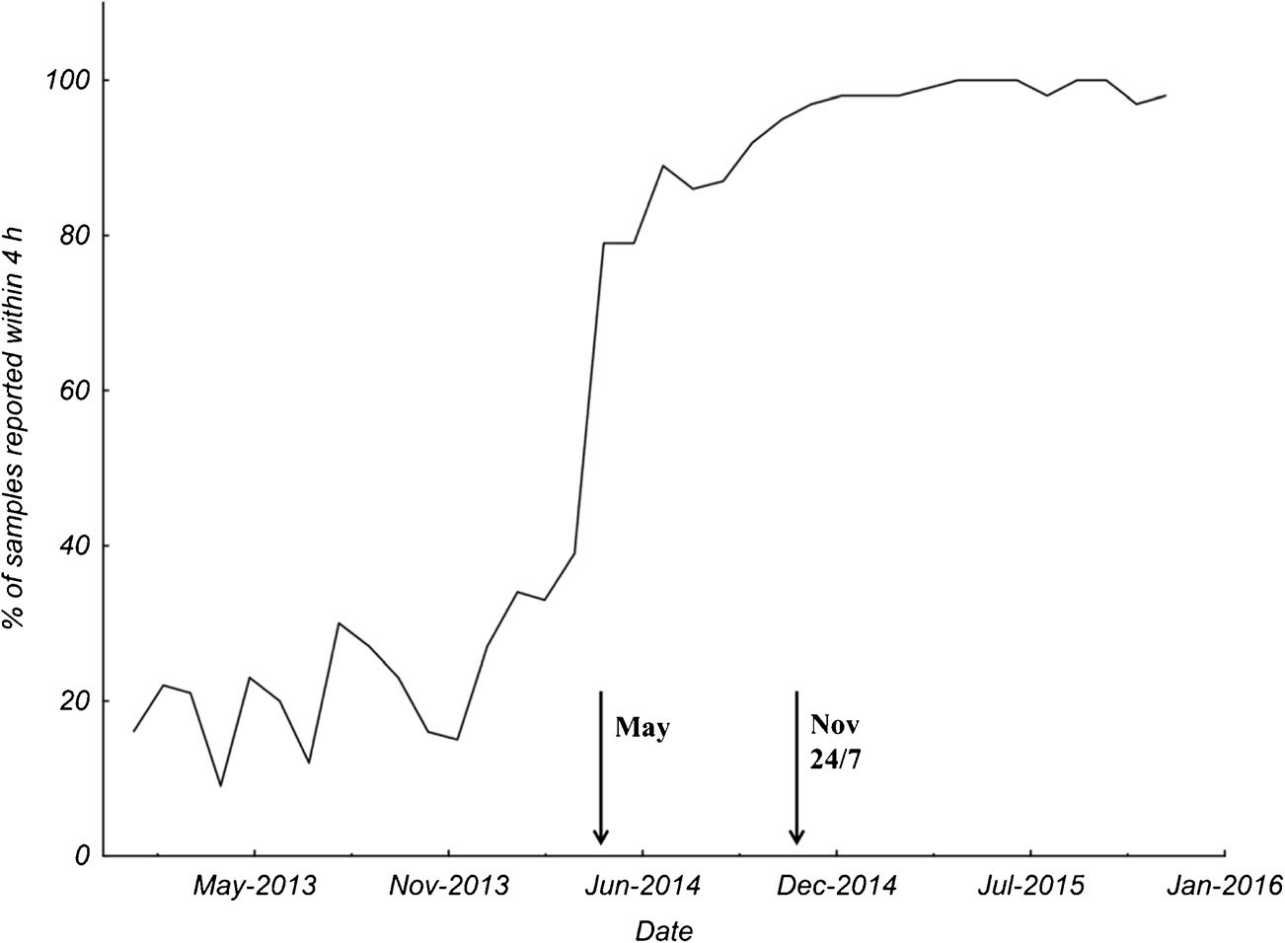
Percentage of test results reported within 4 hours from sample arrival at the laboratory before and after the GeneXpert® Norovirus assay was implemented at the laboratories of clinical chemistry as a 24/7 service at all three hospitals in the county. May 2014: analysis was performed during the daytime with GeneXpert. November 2014: analysis of NoV was implemented as a 24/7 service

The percentage of analytical test results reported within 4 h was nearly 100% after the introduction of the 24/7 service, as compared to 10–30% before the implementation of the new method (Fig. 1).

## Discussion

NoV is the most common cause of gastroenteritis in all age groups globally [15]. A rapid and accurate diagnosis is crucial for appropriate infection prevention measures and reduces the necessity of additional diagnostic procedures.

The results of the Xpert Norovirus assay presented here indicate high performance regarding detection and differentiation of the prominent NoVs genogroups, which has also been shown by others [11]. In fact, the Xpert Norovirus assay seemed to have a higher sensitivity and to be more reliable in detecting both NoV GI and GII than the in-house PCR [12] as well as the third PCR [13, 14]. The samples that showed positive test results only with the Xpert assay had high C_t_ values, indicating a low viral load in the samples or possibly unspecific nucleic acid amplification. However, we think that unspecific PCR products are a less plausible explanation, since all patients had symptoms of acute gastroenteritis.

The median TAT was substantially reduced when single-unit analysis on demand was practicable and the same 24/7 laboratory service could be provided at all three hospitals. Rapid identification of NoV cases facilitates adequate infection control measures, as well as planning of medical staff resources and is likely to reduce the healthcare costs, although we have not studied these economic aspects in this work.

The Xpert Norovirus assay is rapid and easy to use, and requires little hands-on time. Manual interpretation and registration of test results have now been replaced by interpretation by the Xpert software and automatic data transfer from the GeneXpert instrument to the laboratory information system. Single sample analysis reduces TAT since no batching of samples is necessary, and furthermore, it decreases the risk for contamination and mix-up of samples.

Even though we see several advantages in the decentralization of easy-to-use assays, like the Xpert Norovirus assay, to facilities open 24/7, we believe that it is important that specialists with experience from molecular virology maintain the responsibility for validation and follow up of the diagnostic performance, and that in-house PCR methods are available for complementary analysis when required.

In conclusion, we found that the diagnostic performance of the Xpert Norovirus assay was excellent, and since the analytical platform and the ease of performing the test allowed its implementation as a 24/7 service at all hospitals in our county, it has entailed a significant time gain for the patients and the healthcare providers, as well as a more efficient, automated and less stressful work flow in the laboratory.

## Electronic supplementary material

ESM 1 (XLSX 22 kb)
